# A Caps-Ubi Model for Protein Ubiquitination Site Prediction

**DOI:** 10.3389/fpls.2022.884903

**Published:** 2022-05-25

**Authors:** Yin Luo, Jiulei Jiang, Jiajie Zhu, Qiyi Huang, Weimin Li, Ying Wang, Yamin Gao

**Affiliations:** ^1^School of Life Sciences, East China Normal University, Shanghai, China; ^2^School of Computer Science and Engineering, Changshu Institute of Technology, Suzhou, China; ^3^School of Computer Engineering and Science, Shanghai University, Shanghai, China; ^4^School of Computer Science and Engineering, North Minzu University, Yinchuan, China

**Keywords:** protein ubiquitination, site prediction, capsule network, hybrid encoding, plant protection

## Abstract

Ubiquitination, a widespread mechanism of regulating cellular responses in plants, is one of the most important post-translational modifications of proteins in many biological processes and is involved in the regulation of plant disease resistance responses. Predicting ubiquitination is an important technical method for plant protection. Traditional ubiquitination site determination methods are costly and time-consuming, while computational-based prediction methods can accurately and efficiently predict ubiquitination sites. At present, capsule networks and deep learning are used alone for prediction, and the effect is not obvious. The capsule network reflects the spatial position relationship of the internal features of the neural network, but it cannot identify long-distance dependencies or focus on amino acids in protein sequences or their degree of importance. In this study, we investigated the use of convolutional neural networks and capsule networks in deep learning to design a novel model “Caps-Ubi,” first using the one-hot and amino acid continuous type hybrid encoding method to characterize ubiquitination sites. The sequence patterns, the dependencies between the encoded protein sequences and the important amino acids in the captured sequences, were then focused on the importance of amino acids in the sequences through the proposed Caps-Ubi model and used for multispecies ubiquitination site prediction. Through relevant experiments, the proposed Caps-Ubi method is superior to other similar methods in predicting ubiquitination sites.

## Introduction

Ubiquitination is an important post-translational modification of proteins, consisting of the covalent binding of ubiquitin to a variety of cellular proteins. Ubiquitin was discovered in 1975 by Goldstein et al. (1975); it is a small protein composed of 76 amino acids (Wilkinson, 2005). Ubiquitination is the process of covalently binding the lysine of a substrate protein to an ubiquitin molecule, which is catalyzed by a series of enzymes. Three enzymes are involved in this process: E1 activation, E2 conjugation, and E3 ligation. Ubiquitination modification plays a very important role in basic reactions such as signal transduction, diseases, DNA repair, and transcriptional regulation (Hicke, 2001; Pickart, 2001; Haglund and Dikic, 2005; Hicke et al., 2005). Due to the important biological characteristics of ubiquitination, identifying potential ubiquitination sites aids in the understanding of protein regulation and molecular mechanisms. Determining ubiquitination sites based on traditional biological experimental techniques such as mass spectrometry (Peng et al., 2003) and antibody recognition (Gentry et al., 2005) is costly and time-consuming. Therefore, it is necessary to develop a method that can accurately and efficiently recognize protein ubiquitination. In recent years, some calculation methods have been developed to predict potential ubiquitination sites. Huang et al. (2016) used amino acid composition (AAC), a position weighting matrix, amino acid pair composition (AAPC), a position-specific scoring matrix (PSSM), and other information to develop a predictor called UbiSite using a support vector machine (SVM). Nguyen et al. (2016) used an SVM to combine three kinds of information: AAC, evolution information, and AAPC to develop a predictor. Qiu et al. (2015) developed a new predictor called “iUbiq-Lys” to apply to sequence evolution information and a gray system model. Chen et al. (2013) also applied SVM to build a UbiProber predictor. Wang et al. (2017b) introduced physical–chemical attributes into an SVM to develop the ESA-UbiSite predictor. Radivojac et al. (2010) developed the predictor UbPred using a random forest algorithm. Lee et al. (2011) developed UbSite using efficient radial basis functions. All of those machine learning-based methods and predictors have promoted the development of ubiquitination site prediction research and achieved good prediction performance. However, most of them rely on artificial feature selection, which may lead to imperfect features (Wang et al., 2017b), and their datasets are small despite the large volume of accumulated biomedical data.

Deep learning, the most advanced machine learning technology, can handle large-scale data well. It has multilayer networks and nonlinear mapping operations, which can fit the complex structure of data well. In recent years, deep learning has been developed rapidly (Wang et al., 2017a) and has been successfully applied in various fields of bioinformatics (Sun et al., 2017; Shaw et al., 2019). In using evolutionary information on proteins, there are predictions of ATP-binding sites using two-dimensional convolutional neural networks and position-specific scoring matrices (Kusuma and Ou, 2019; Le et al., 2019). Some methods based on deep learning have been used for ubiquitination site identification. For example, Fu et al. (2019) applied one-hot and composition of k-spaced amino acid pairs encoding methods to develop DeepUbi with text-CNN. Liu et al. (2021) used deep transfer learning methods to develop the DeepTL-Ubi predictor for multispecies ubiquitination site prediction. He et al. (2018) established a multimodel predictor using one-hot, physical–chemical properties of amino acids, and a PSSM.

Although various ubiquitination site predictors and tools have been developed, there are still some limitations, and their accuracy and other performance elements must be further improved. In this study, a deep learning model, “Caps-Ubi,” is proposed that uses a capsule network for protein ubiquitination site prediction. In Caps-Ubi, the protein fragments are first passed through one-hot and amino acid continuous methods to encode them. Then three convolutional layers and the capsule network layer are used as a feature extractor to obtain the functional domains in the protein fragments and finally to obtain the prediction results. Relative to existing tools, the prediction performance of Caps-Ubi is a significant improvement. Researchers can use the predictor to select potential ubiquitination candidate sites and perform experiments to verify them, which will reduce the range of protein candidates and save time.

## Materials and Methods

### Benchmark Dataset

The ubiquitination dataset came from the largest online protein lysine modification database, PLMD 3.0, which contains 20 protein lysine modifications. The database has 53,501 proteins and 284,780 protein lysine modification sites, including 25,103 proteins and 121,742 ubiquitination sites. To eliminate errors caused by homologous sequences, we used CD-HIT (Huang et al., 2010) to filter out homologous sequences with sequence similarities greater than 40%. We obtained 12,100 proteins and 54,586 ubiquitination sites after filtering, which were used as a positive sample set. Based on these 12,100 protein sequences with annotation information, 427,305 non-ubiquitinated sites were extracted from the proteins as a negative sample set for model training. These negative sample sequences were filtered by CD-HIT-2D, and the sequences that were more than 50% homologous to the positive samples were filtered out, and 320,083 non-ubiquitinated sites were obtained after filtering. Since there are only 54,586 positive sample sets and 320,083 negative sample sets, the difference between positive and negative samples is nearly 8 times. If such a data distribution is used to input the model for training, the model will not be able to fully learn the data features of the positive samples, and the obtained prediction results will tend to be in the negative sample set. To establish a balanced training model, we randomly selected the same data as the positive sample set and selected 90% of it as the training and validation sets and 10% as the independent test set. Finally, 53,999 data points on ubiquitination sites and 50,315 data points on nonubiquitination sites were obtained. The final data division is shown in Table 1.

**Table 1:** Data on protein ubiquitination sites.

Dataset	No. of positive data	No. of negative data
Training	44,214	44,214
Validation	4,913	4,913
Testing	5,459	5,459

### Input Sequence Coding

The coding method directly determines the quality of its prediction results; a good feature can extract the correlation between the ubiquitination feature and the targets from peptide sequences (Plewczynski et al., 2005). After encoding the protein sequence, the sequence information is converted into digital information, and then deep learning is performed on it. In this study, two methods were used to encode the amino acid sequence around the protein ubiquitination site; namely, one-hot encoding and amino acid continuous encoding.

#### One-Hot Encoding

**Figure 1:**
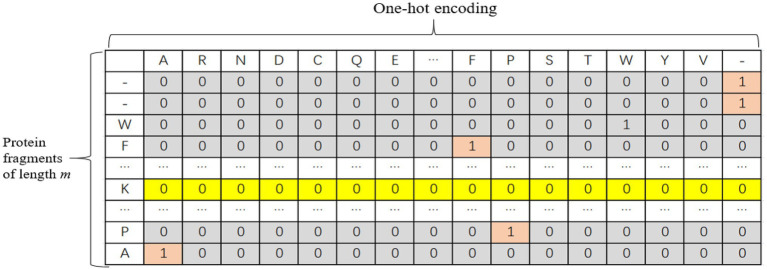
Schematic diagram of one-hot encoding of protein fragments.

The one-hot encoding method was adopted for protein fragments. There are 20 common amino acids. In this paper, each protein fragment is coded into an m × k two-dimensional matrix, where *k* represents the size of the dictionary, and *m* represents the number of amino acids in each sequence, i.e., the length of the input sequence. When the length of the input sequence does not reach the window length, padded with “-” to the left or right of the protein fragment and treat it as another amino acid. Therefore, each amino acid is actually represented by a one-dimensional vector with a length of 21. Only the position corresponding to this amino acid is 1, and the other positions are 0. The one-hot encoding is shown in Figure 1.

#### Continuous Coding of Amino Acids

The continuous amino acid coding method (Venkatarajan and Braun, 2001) was proposed by Venkatarajan; the coding uses 237 physical–chemical properties to quantitatively characterize 20 amino acids. They used five main components to characterize the changes in 237 physico-chemical properties of amino acids. In this paper, each amino acid is represented by a six-dimension vector, wherein the first five-dimension represents the five principal components as shown in Table 1 of Venkatarajan and Braun (2001), and the last one-dimension represents the gap in the input protein fragment with a length of *m*. The gap is represented by a dash“-,” meaning that when the sequence length does not reach the window length, the bit is coded as 1; otherwise, it is 0. Finally, each protein fragment is coded into an *m* × 6 two-dimension matrix. This continuous coding scheme can comprehensively consider the physical and chemical properties of protein amino acids and has a smaller dimension than that of one-hot coding. The smaller input dimension will lead to a relatively simple network structure, which is beneficial to avoid overfitting.

### Capsule Network

In a CNN, the pooling layer can extract valuable information from the data, but some location information is lost (Dombetzki, 2018). Additionally, a CNN outputs scalar values in neurons, and the information represented by scalar neurons is limited and cannot reflect the spatial position relation of the internal features of the neural network. To solve the problems of scalar neurons, in 2017 Hinton proposed a deep learning architecture called a capsule network (Sabour et al., 2017). The primary building module of a capsule network is the capsule (Hinton et al., 2011), which is a set of neuron vectors. The length of the capsule represents the probability of the existence of an entity; the longer the capsule is, the greater the probability, and the direction of the capsule represents the state of the entity. The capsule network provides a unique and powerful deep learning building block that can better model the complex relationships within a neural network. A CNN uses scalar input activation functions, such as the rectified linear activation function ReLU, a sigmoid, and a tanh, and the capsule network uses an activation function called a squash. The calculation equation is


(1) $$v_{j}=\frac{{\left\|s_{j}\right\|}^{2}}{1+\|s_{j}\|{}^{2}}\frac{s_{j}}{\left\|s_{j}\right\|}$$

where $v_{j}$ is the output of capsule j, and *s_j_* is the weighted sum of the input vectors of capsule j . This function compresses the vector length to the interval [0,1], which can be regarded as a kind of compression and reallocation of the vector length. In addition to the first-layer capsule network, the input of the capsule *s_j_* is obtained by the weighted sum of the prediction vector ( ${\hat{u}}_{j|i}$ ) located in the lower-layer capsule, and the prediction vector ( ${\hat{u}}_{j|i}$ ) is passed through the lower layer. The capsule is calculated by multiplying its output $\left(u_{i}\right)$ and the weight matrix ( $w_{ij}$ ):


(2) $$s_{j}=\underset{i}{\sum }c_{ij}{\hat{u}}_{j|i}\text{,}$$

(3) $${\hat{u}}_{j|i}=w_{ij}u_{i}\text{,}$$

where $c_{ij}$ is the coupling coefficient, which is obtained by a softmax transformation from $b_{ij}$ ; its calculation equation is


(4) $$c_{ij}=\frac{\exp\left(b_{ij}\right)}{\sum_{k}\exp\left(b_{ik}\right)}\text{.}$$

In Eq. (4), the sum of the coupling coefficients of all capsules and capsule i in the previous layer is 1. The coupling coefficient is obtained through a dynamic routing mechanism; the pseudocode is shown in Table 2.

**Table 2:** The pseudocode of a dynamic routing mechanism.

ROUTING ( ${\hat{u}}_{j|i}$ , r, l)
**Input:** ${\hat{u}}_{j|i}$ , *r, l*
**Output:** *v_j_*
for all capsules *i* in layer *l* and capsules *j* in layer (*l* + 1): *b_ij_*←0.
for *r* iterations do:
for all capsules *i* in layer *l*: *c_i_*←softmax ( $b_{i}$ ) end for
for all capsules *j* in layer (*l* + 1): $s_{j}\leftarrow \Sigma c_{ij}{\hat{u}}_{j|i}$
*v_j_*←squashing ( $s_{j}$ ) end for
for all capsules *i* in layer *l* and capsules *j* in layer (*l* + 1): *b_ij_*←*b_ij_*+ ${\hat{u}}_{j|i}.v_{j}$ end for return $v_{j}$ end for
end for

The loss function of the capsule network is the margin loss function, and the calculation equation is


(5) $$L_{k}=T_{k}\max{\left(0,m^{+}-\|V_{k}\|\right)}^{2}+\lambda \left(1-T_{k}\right)\max{\left(0,\|V_{k}\|-m^{-}\right)}^{2}\text{,}$$

where K is the number of categories, $T_{K}$ is the real label ubiquitinated to 1 and nonubiquitinated to 0, and $\left\|V_{k}\right\|$ is the output length of the *k*th capsule, which is the probability of predicting the *k*th class. The boundary $m^{+}$ is 0.9, which is a penalty for false-positives, and the lower boundary $m^{-}$ is 0.1, which is a penalty for false negatives. λ is a proportional coefficient of 0.5, which is used to control the loss caused when some categories do not appear to prevent the capsule vector length of all categories from being reduced in the early stage of training, and the total loss is the sum of the losses of Kcategories .

### Architecture Design

**Figure 2:**
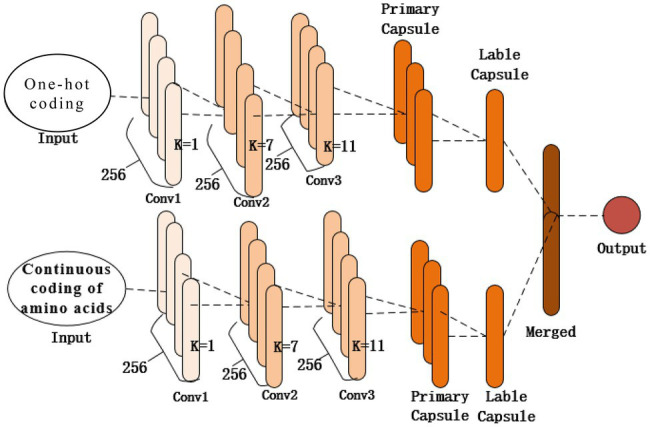
Network structure of the proposed model.

As shown in Figure 2, the structure of the proposed model contains two identical subnetworks that process one-hot and amino acid continuous encoding modes. After training in their respective network model, the two models merge the features as the final output. Each subnetwork consists of the same three 1D convolutional layers (Conv1, Conv2, and Conv3) and a capsule network layer. The first convolutional layer (Conv1) of the network is a 1D convolution kernel, which comprises 256 convolution kernels with a size of 1 and a step size of 1 that use the ReLU activation function. A convolution kernel with a length of 1 first appears in the Network in Network (Lin et al., 2013); a convolution kernel with a length of 1 can reduce the complexity of the model and can make the network deeper and wider. Applied in this study, it acts as a feature filter and can pool features in two encoding modes. The second convolutional layer, Conv2, is a conventional convolutional layer with 256 1D convolution kernels with a length of 7 and a step size of 1, which functions as a local feature detector to extract the protein sequence input and convert it to corresponding local features. Conv2 is understood as the functional domain characteristics of the protein, and its output is used as the input of the next layer, Conv3. The third convolutional layer, Conv3, has 256 1D convolution kernels with a size of 11 and a step size of 1. The activation function used is ReLU and a dropout mechanism with a random deletion rate of 0.3. The dropout mechanism is used to prevent the model from overfitting and to increase the generalization ability of the model. These two convolutional layers are used to increase the feature representation ability of the capsule network and convert the original features of protein fragments into more advanced and abstract features. Then the local features of Conv2 are used as the input of the PrimaryCapsule network layer. The dimension of each capsule in PrimaryCapsule is 8, the step size is 1, the convolution kernel length is 20, and the squash activation function is used. The last layer of LabelCapsule is a capsule with a dimension of 10, which is used to represent the two states of the input protein fragment: the input sequence is the ubiquitination site or non-ubiquitination site, and finally the output of the two subnetworks is merged as the final prediction result.

### Model Training

For model training, we used the Adam (Kingma and Ba, 2014) optimization algorithm. Adam automatically adjusts the learning rate of the parameters, improve the training speed, and improve the stability of the model. The learning rate was 0.003, the first-order estimated exponential decay rate was 0.9, and the exponential decay rate estimated by the second moment was 0.999. The dynamic routing mechanism was consistent with that in the original paper (Dombetzki, 2018). The number of routing iterations was 3, and the boundary loss function was used as the loss function of the model. The boundary loss function form is shown in Eq. (5). Since the data samples of the input model are about 100,000, and due to the limitation of computing resources, all the data cannot be input into the model for training at one time. The size of each data block (batch_size) is 124. The training of the entire training set is completed once called an epoch, and the early stopping mechanism is used during the training process. When the loss of the validation set does not decrease after 10 epochs, the entire training process is terminated, and the final model tends to be stable at 50 epochs, that is, the epoch of this model is 50. The deep learning framework used by this model was Keras 2.1.4. Keras is a highly modular deep learning framework based on Theano and written in Python; it supports both CPU and GPU. The programming language was Python 3.5, and the model was trained and tested on a Windows 10 system equipped with an Nvidia RTX 2060 GPU.

## Results

### Model Evaluation and Performance Indicators

A confusion matrix is a visual display tool used to evaluate the quality of classification models. Each row of the matrix represents the actual condition of the sample, and each column represents the sample condition predicted by the model. There are four values in the matrix, as shown in the following equations, where FN is the number of false negatives, FP is the number of false-positives, TN is the number of true negatives, and TP is the number of true positives. The following indicators based on the confusion matrix are usually used to evaluate the prediction of the model performance:


(6) $$S_{n}=\frac{TP}{FN+TP}\text{,}$$

(7) $$S_{p}=\frac{TN}{TN+FP}\text{,}$$

(8) $$A\mathrm{cc}=\frac{TP+TN}{TP+TN+FP+FN}\text{,}$$

(9) $$MCC=\frac{TP\times TN-FN\times FP}{\sqrt{\left(TP+FN\right)\left(TN+FP\right)\left(TP+FP\right)\left(TN+FN\right)}}\text{,}$$

*S_n_* stands for sensitivity, which is the evaluation of the prediction performance of negative samples; *S_p_* is the specificity, which is the evaluation of the prediction performance of positive samples; Acc is the accuracy, which is the evaluation of the accuracy of the model; and MCC is Matthew’s correlation coefficient, which is the overall evaluation of the model. The receiver operating characteristic (ROC) curve and the area under the curve (AUC) for the ROC curve are usually used to evaluate the pros and cons of binary classifiers. AUC is defined as the area under the ROC curve bounded by the coordinate axis. A classifier with a larger AUC value has a higher accuracy rate.

### Experimental Results

**Figure 3:**
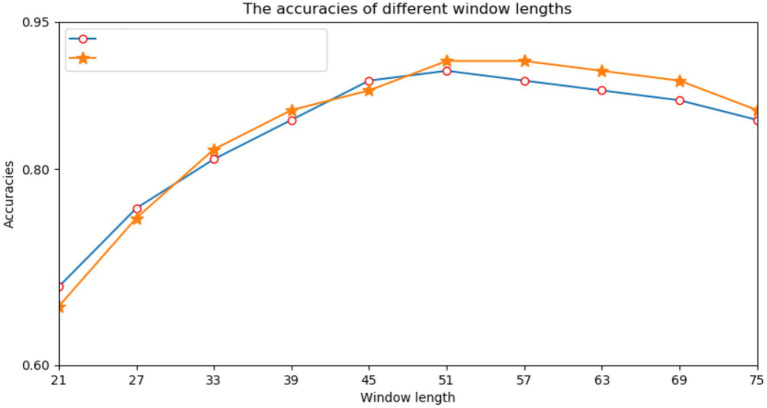
Accuracy of the verification set for various window lengths.

First, we performed many experiments on the selection of the window size of protein fragments. Because the correlation information between amino acids had a direct effect on the prediction results, we needed to determine an appropriate window size. Previous studies have directly used empirical values such as 21, 33, or 49. However, different data models and classifiers tend to have different window sizes. Therefore, a window length of *n* was selected from a range of 21–75, and we performed a series of experiments using different window lengths. For each window length, we encoded all training data into two input modes and trained their respective subnetworks. According to the prediction results of the validation set, we selected each appropriate window size. Figure 3 shows the performance of various window sizes in one-hot and amino acid continuous encoding modes.

In Figure 3, the abscissa represents the window length, and the ordinate represents the accuracy of the model. Figure 3 shows that when the window length was 51, the two encoding modes had the highest accuracy. Therefore, we set the window length of this model to 51. That is, take lysine *K* as the center, give 25 amino acids to the left and right, and fill in with “-” when the sequence length does not reach the window length.

To compare the performance of the model under different encoding schemes, we compared the capsule network and the CNN to similar hierarchical structures of capsule networks and the same training set size. The CNN structure replaced only the PrimaryCapsule layer with the Conv3 layer. We set the LabelCapsule layer to a 128 × 1 fully connected layer. The comparison results are shown in Table 3.

**Table 3:** Comparison of various coding schemes.

Feature	Model	Acc (%)^1^	*S_n_* (%)^2^	*S_p_* (%)^3^	AUC^4^	MCC^5^
One-hot	CapsNet	89.51	93.70	85.31	0.96	0.80
	CNN	84.93	86.39	82.93	0.93	0.70
Amino acid continuous	CapsNet	90.06	91.88	88.23	0.96	0.80
	CNN	83.83	85.25	82.41	0.91	0.68
One-hot and amino acid continuous	CapsNet	90.47	93.66	87.27	0.96	0.81
	CNN	84.67	82.62	86.72	0.93	0.70

1 Accuracy of the model

2 Sensitivity of the model.

3 Specificity of the model.

4 Area under curve.

5 Matthew’s correlation coefficient.

**Figure 4:**
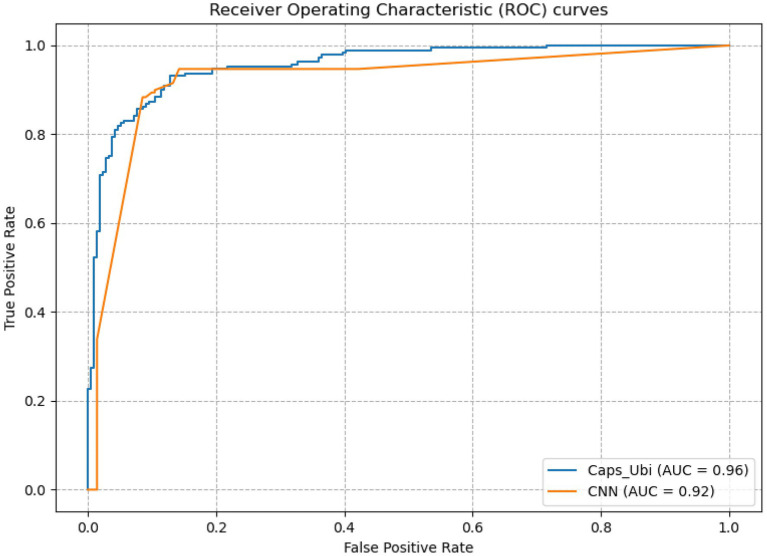
Receiver operating characteristic curve of Caps-Ubi and CNN on the test set.

From Table 3, it can be concluded that the capsule network’s accuracies were 5.39, 7.43, and 6.85% percentage points higher than those of CNN under the one-hot, amino acid continuous, and combined one-hot and amino acid continuous types, indicating that the capsule network internally expressing the hierarchical relation modeling aspect has more advantages than CNN. Among them, the performance under the combined one-hot and amino acid continuous encoding modes is the best on the capsule network: this proposed Caps-Ubi model achieved an accuracy, sensitivity, specificity, area under curve, and Matthew’s correlation coefficient of 91.23%, 93.11%, 89.34%, 0.96, and 0.83, respectively. The proposed Caps-Ubi was obtained from balanced data. The ROC curve of Caps-Ubi on the test set is shown in Figure 4, demonstrating that it was very close to the real situation.

When we used balanced data to train the model on an experimentally verified ubiquitination dataset and a nonubiqui tination dataset (Fu et al., 2019), the ratio of positive peptides and negative peptides was 1:8, so we tested Caps-Ubi using natural-distribution data. The test results are shown in Table 4. According to the test results, the performance was slightly worse than that under the balanced data.

**Table 4:** Results of testing Caps-Ubi under natural-distribution data.

Protein fragment	Acc (%)^1^	*S_n_* (%)^2^	*S_p_* (%)^3^	AUC^4^	MCC^5^	Positive–negative ratio
1,000	53.75	0.08	0.99	0.70	0.19	1:8
10,000	53.30	0.12	0.95	0.59	0.12	1:8

1 Accuracy of the model.

2 Sensitivity of the model.

3 Specificity of the model.

4 Area under curve.

5 Matthew’s correlation coefficient.

### Comparison to Other Methods

In the past 10 years, many researchers have contributed to the prediction and research of protein ubiquitination sites. Therefore, we compared the proposed model to other sequence-based prediction tools. The corresponding data and results are shown in Table 5, which shows that the performance of the Caps-Ubi model exceeded that of the best-performing deep learning model DeepUbi and several other prediction models. Among them, machine learning-based predictors improve accuracy by adding new features. In this study, we propose a deep learning model Caps-Ubi, which has good performance on large datasets and learns deeper features. The accuracy, sensitivity, specificity, area under the curve, and Matthew’s correlation coefficient of Caps-Ubi were 2.36, 3.31, 1.24, 0.05, and 0.05 respectively, several percentage points highe r than those of DeepUbi.

**Table 5:** Proposed Caps-Ubi compared with other methods.

Predictor	Acc (%)^1^	*S_n_* (%)^2^	*S_p_* (%)^3^	AUC^4^	MCC^5^
UbiPred (Lee et al., 2011)	84.44	83.44	85.43	0.85	0.69
UbSite (Lee et al., 2011)	74.5	65.5	74,8	–	–
CKSAAP_UbSite (Chen et al. 2011)	73.4	69.85	76.96	0.81	0.47
UbiProber (Chen et al., 2013)	–	37.0	90.0	0.77	0.63
iUbiq-Lys (Qiu et al., 2015)	82.14	80.56	99.39	–	0.50
DeepUbi (Fu et al., 2019)	88.98	89.8	88,10	0.91	0.78
Caps-Ubi	91.34	93.11	89.34	0.96	0.83

1 Accuracy of the model.

2 Sensitivity of the model.

3 Specificity of the model.

4 Area under curve.

5 Matthew’s correlation coefficient.

## Conclusion

In this study, a new deep learning model for predicting protein ubiquitination sites is proposed, using one-hot and amino acid continuous coding modes. We used the largest available protein ubiquitination site dataset, and the experimental results above verify the efficacy of this model. Operation of the model involves four main steps: encoding protein sequences, constructing convolutional layers, constructing a capsule network layer, and constructing an output layer. The capsule network introduces a new building block for deep learning. Relative to CNN, the capsule network, which uses a dynamic routing mechanism to update parameters, requires more training time, but the time required for prediction is similar. The capsule network can also characterize the complex relationships among amino acids in various sequence positions and can be used to explore the internal data distribution related to biochemical significance. The proposed Caps-Ubi prediction tool will facilitate the sequence analysis of ubiquitination and can also be used to identify other post-translational modification sites in proteins. In the future, we will study other features that may better extract sample attributes to construct deeper models.

## Data Availability Statement

The original contributions presented in the study are included in the article/supplementary files, further inquiries can be directed to the corresponding authors.

## Author Contributions

YL: methodology, and writing—review and editing. JJ: conceptualization, and review and editing. JZ: writing and editing. WL: conceptualization and supervision. YW: investigation. YG: data curation. All authors contributed to the article and approved the submitted version.

## Funding

This project is supported by the National Key R&D Program of China (2018YFE0194000), National Nature Science Foundation of China (grant no. 61762002), and National Statistical Science Research Project (2020LY074).

## Conflict of Interest

The authors declare that the research was conducted in the absence of any commercial or financial relationships that could be construed as a potential conflict of interest.

## Publisher’s Note

All claims expressed in this article are solely those of the authors and do not necessarily represent those of their affiliated organizations, or those of the publisher, the editors and the reviewers. Any product that may be evaluated in this article, or claim that may be made by its manufacturer, is not guaranteed or endorsed by the publisher.
